# Evaluation of Refractive Predictive Accuracy in Intraocular Lens Power Calculations: A Comparative Study of Swept-Source Optical Coherence Tomography and Optical Low-Coherence Interferometry

**DOI:** 10.3390/jcm14041201

**Published:** 2025-02-12

**Authors:** Leila Al Barri, Nadina Mercea, Yasar Ionela-Iasmina, Mihnea Munteanu, Horia T. Stanca

**Affiliations:** 1 Ophthalmology Department, “Victor Babes” University of Medicine and Pharmacy, 300041 Timisoara, Romania; leila.albarri@umft.ro (L.A.B.); nadina.mercea@umft.ro (N.M.); ionela.yasar@umft.ro (Y.I.-I.); 2 Oftalmo Sensory-Tumor Research Center—ORL (EYE-ENT), Ophthalmology Department, “Victor Babes” University of Medicine and Pharmacy, 300041 Timisoara, Romania; 3 Ophthalmology Department, “Carol Davila” University of Medicine and Pharmacy, 050474 Bucharest, Romania; horia.stanca@umfcd.ro

**Keywords:** cataract surgery, intraocular lens (IOL), biometry, swept-source optical coherence tomography (SS-OCT), optical low-coherence interferometry (OLCI), refractive predictive accuracy, axial length measurement, ocular biometry, refractive outcomes, IOL power calculation

## Abstract

**Background/Objectives:** Precise intraocular lens (IOL) power calculations are essential for achieving optimal refractive outcomes in cataract surgery. This study compares the predictive accuracy of swept-source optical coherence tomography (SS-OCT) and optical low-coherence interferometry (OLCI) in biometry measurements and refractive outcomes. **Methods:** This retrospective study included 170 eyes from 102 patients undergoing cataract surgery. Biometry was performed using Argos^®^ (MOVU Inc., Komaki, Japan) (SS-OCT) and Aladdin^®^ (Topcon Corp., Tokyo, Japan) (OLCI), measuring axial length (AL), anterior chamber depth (ACD), lens thickness (LT), white to white (WTW), and keratometry. **Results:** Postoperative outcomes, including uncorrected and corrected distance visual acuity (UDVA, CDVA), spherical equivalent (SE), and refractive error, were assessed at one and six months. Predictive accuracy was evaluated by mean error (ME), mean absolute error (MAE), median absolute error (MedAE), and the percentage of eyes within ±0.25 D to ±1.00 D of predicted SE. **Conclusions:** Both technologies achieved high refractive accuracy, with 97.7% (SS-OCT) and 97.2% (OLCI) of eyes within ±1.00 D of target SE. SS-OCT demonstrated superior axis alignment, while OLCI provided enhanced postoperative SE. Significant differences were observed in LT (*p* = 0.030) and ACD (*p* = 0.009). Postoperative UDVA of 20/20 or better was achieved in 98% of SS-OCT eyes and 100% of OLCI eyes. SS-OCT and OLCI provide comparable refractive outcomes and high reliability in cataract surgery.

## 1. Introduction

Cataract surgery is among the most frequently performed surgical procedures globally, providing significant improvements in patients’ visual function and quality of life. Advances in biometry have enabled precise measurements of ocular parameters essential for accurate IOL power calculations. These improvements have increased not only the predictability of refractive outcomes but also patient expectations, particularly among those seeking spectacle independence through multifocal (MF) or extended depth-of-focus (EDOF) IOLs [1,2,3].

Errors in ocular biometry can result in refractive surprises, leading to unsatisfactory visual outcomes. Traditional ultrasound biometry, once the standard, has largely been replaced by optical methods due to their non-contact nature and superior resolution [4,5]. Technologies such as partial coherence interferometry (PCI) and optical low-coherence interferometry (OLCI) have provided significant advancements in axial length (AL) and anterior segment measurements [6,7]. More recently, swept-source optical coherence tomography (SS-OCT) has emerged as a promising technology, offering improved imaging depth and precision [8,9,10].

SS-OCT technology uses a tunable laser with a longer wavelength, typically around 1055–1060 nm, enabling superior tissue penetration and high-resolution imaging of ocular structures. Devices such as the Argos^®^ (MOVU Inc., Komaki, Japan) biometer influence this technology to provide precise measurements of AL, anterior chamber depth (ACD), lens thickness (LT), and white to white (WTW), even in the presence of dense cataracts [2,6,9]. The Argos also incorporates a “segmental refractive index” approach, which assigns separate refractive indices to each segment of the eye, enhancing the accuracy of AL measurements in long or short eyes [1,11].

In contrast, OLCI-based devices like the Aladdin^®^ (Topcon Corp., Tokyo, Japan) biometer utilize a shorter wavelength for optical biometry combined with Placido-disk corneal topography, allowing simultaneous measurement of AL, ACD, keratometry (K), corneal astigmatism, and higher-order aberrations [4,6]. While OLCI technology excels in its precision and ability to integrate biometry with topographic data, its efficacy may be reduced in eyes with dense media opacities compared to SS-OCT [5,11]. However, the combination of biometry and topographic data provides clinicians with a comprehensive understanding of the ocular anatomy, aiding in IOL selection and surgical planning [3,8].

Despite the widespread use of both SS-OCT and OLCI devices, comparative studies examining their performance in predicting refractive outcomes remain limited. Research comparing SS-OCT biometry, such as that provided by the Argos^®^, and OLCI biometry, like the Aladdin^®^, is essential for identifying the specific advantages and limitations of each technology [7,9,10]. SS-OCT’s superior penetration through dense cataracts makes it particularly valuable in challenging cases, while OLCI’s detailed corneal analysis can aid in addressing complex refractive needs [3,5].

This study aims to evaluate the predictive accuracy of IOL power calculations using SS-OCT and OLCI technologies. By comparing preoperative biometry, surgical planning data, and postoperative outcomes, this research seeks to clarify the strengths of these technologies.

## 2. Materials and Methods

### 2.1. Study Design, Ethics, and Informed Consent

This retrospective, observational study evaluated refractive and visual outcomes following successful cataract surgery. The study was conducted at the Ophthalmology Department of “Victor Babes” University of Medicine and Pharmacy in Timisoara, Romania, over a period spanning from January 2022 to June 2024.The study adhered to the principles outlined in the Declaration of Helsinki. Due to the retrospective nature of the study, the institutional ethics committee waived specific approval requirements. Furthermore, the informed consent for surgery obtained from all participants explicitly included consent for data use in research studies. Patient confidentiality was maintained throughout the study.

### 2.2. Subjects and Inclusion/Exclusion Criteria

Subjects included were those undergoing uneventful cataract surgery with intraocular lens (IOL) implantation. The allocation of biometry measurements to either SS-OCT or OLCI was determined through a non-randomized process based on clinical workflow and device availability, ensuring a balanced representation of cases for comparative analysis.

Inclusion criteria consisted of: (1) Healthy eyes with no history of ocular trauma. (2) Absence of prior ocular surgery. (3) No systemic diseases impacting ocular health. (4) Preoperative corrected visual acuity (CDVA) better than 0.4 logMAR. (5) Regular corneal astigmatism. Exclusion criteria encompassed: (1) Any history of ectatic corneal diseases. (2) Severe dry eye. (3) Retinal pathologies. (4) Prior refractive surgery.

### 2.3. Procedure

All patients underwent a comprehensive preoperative ophthalmic examination. The cataracts were classified preoperatively according to the Lens Opacities Classification System III (LOCS III), providing a standardized assessment of lens opacification. The preoperative variables assessed included AL, ACD, LT, WTW, keratometry (flat, steep and axis), and visual acuity, which was recorded using a Snellen chart and converted to logMAR for analysis. The IOL power calculations were performed using the Barrett Universal II formula, renowned for its precision across diverse ocular biometry profiles. Cataract surgery was performed by experienced surgeons using a 2.2 mm clear corneal incision and two 1.0 mm side ports. The Verion™ Image Guided System (Alcon Laboratories, Inc., Fort Worth, TX, USA) was employed to assist with the alignment of toric IOLs and the placement of incisions. Postoperative assessments were conducted at one month and six months, including uncorrected and corrected distance visual acuity (UDVA and CDVA), subjective refraction, and spherical equivalent (SE).

### 2.4. Biometers

The biometry measurements were conducted using two devices. The SS-OCT measurements were obtained with Argos^®^ (MOVU Inc., Komaki, Japan), which uses a 1060 nm light source and segmental refractive indices (cornea: 1.376; aqueous and vitreous: 1.336; lens: 1.410) for biometry. The final biometric values were derived from the average of three consecutive measurements. The OLCI measurements were obtained using Aladdin^®^ (Topcon Corp., Tokyo, Japan), which employs an 830 nm light source and a Placido disk for keratometry. Both devices’ parameters measured included keratometry, AL, LT, ACD, and WTW. Three consecutive measurements were taken, and the averages were used for analysis.

### 2.5. Refractive Prediction Calculations

Refractive prediction calculations included the differences between implanted and suggested IOL power in terms of sphere, astigmatism, and axis. The mean error (ME) was defined as the postoperative SE minus the predicted SE, with positive values indicating hyperopic outcomes and negative values indicating myopic outcomes. The mean absolute error (MAE) represented the absolute deviation from the predicted SE, while the median absolute error (MedAE) was the median of absolute differences. Additionally, the percentage of eyes within ±0.25 D, ±0.50 D, ±0.75 D, and ±1.00 D of predicted SE was calculated for both groups.

### 2.6. Statistical Analysis

Statistical analyses were performed using SPSS version 29 (IBM Corp., Armonk, NY, USA). The normality of data was assessed using the Shapiro–Wilk test. For normally distributed data, independent sample *t*-tests were applied, while the Mann–Whitney U test was used for non-normally distributed data. Standard refractive outcomes, as defined by Waring and colleagues [12,13,14,15,16,17], were calculated to evaluate predictive accuracy. To account for the sample size disparity, bootstrapping (10,000 iterations) was used to validate result stability. Normality was assessed before applying appropriate statistical tests, and sensitivity analyses confirmed that findings were not driven by sample size differences. A sensitivity analysis was performed to assess the impact of outliers, confirming that these values, influenced by the preoperative state of the eyes (e.g., prior refractive surgery), did not significantly affect the overall results or conclusions. Results were considered statistically significant at *p* < 0.05.

## 3. Results

The analysis included a total of 102 patients, corresponding to 170 eyes, with 133 eyes in the SS-OCT group and 37 in the OLCI group. Regarding astigmatism types, the SS-OCT group showed 52 eyes with with-the-rule (WTR) astigmatism (39.1%), 58 with against-the-rule (ATR) astigmatism (43.6%), and 23 with oblique astigmatism (17.3%). In the OLCI group, 20 eyes had WTR astigmatism (54.1%), 14 had ATR astigmatism (37.8%), and three had oblique astigmatism (8.1%). For IOL cylindricity, the SS-OCT group included 29 spherical lenses (21.8%) and 104 toric lenses (78.2%), while the OLCI group had 26 spherical lenses (70.3%) and 10 toric lenses (27.0%). The IOL models used were Alcon AcrySof IQ [SN60WF] for spherical lenses and Alcon AcrySof Toric [SN6ATx] for toric lenses.

Table 1 summarizes the preoperative characteristics of the study population, including demographics, refractive parameters, visual acuity, and ocular anatomical metrics. The data, presented as means with standard deviations and ranges, show no significant differences between the SS-OCT and OLCI groups across most variables (*p* > 0.05), except for lens thickness (*p* = 0.030) and anterior chamber depth (*p* = 0.009), where statistically significant differences were observed. This table establishes the comparability of the groups while highlighting subtle differences in specific anatomical metrics.

Table 2 summarizes the surgical data, including IOL spherical and astigmatic powers, axis, and differences between implanted and suggested values. It also includes the ME, MAE, and MedAE for comparisons of expected versus achieved SE at 1 and 6 months postoperatively. Additionally, it reports the percentage of eyes within ±0.25 D, ±0.50 D, ±0.75 D, and ±1.00 D for refractive prediction accuracy at 6 months. While most variables showed no significant differences between the SS-OCT and OLCI groups, statistically significant differences were observed in the difference between the implanted and suggested spherical power (*p* = 0.002) and astigmatic power (*p* = 0.035). This table highlights the role of surgical planning and its impact on refractive outcomes, as well as the high predictability of refractive outcomes for both groups.

Table 3 summarizes the postoperative outcomes at 1 and 6 months, including intraocular pressure (IOP), refractive parameters (sphere, cylinder, axis, and SE), and visual acuity measures UDVA and CDVA. Most variables showed no statistically significant differences between the SS-OCT and OLCI groups (*p* > 0.05). However, a marginally significant difference in cylinder at 6 months was observed (*p* = 0.048). This result with marginal significance should be interpreted with caution. This table provides a detailed comparison of early and intermediate postoperative outcomes and highlights the minor differences between the two biometry technologies.

**Figure 1:**
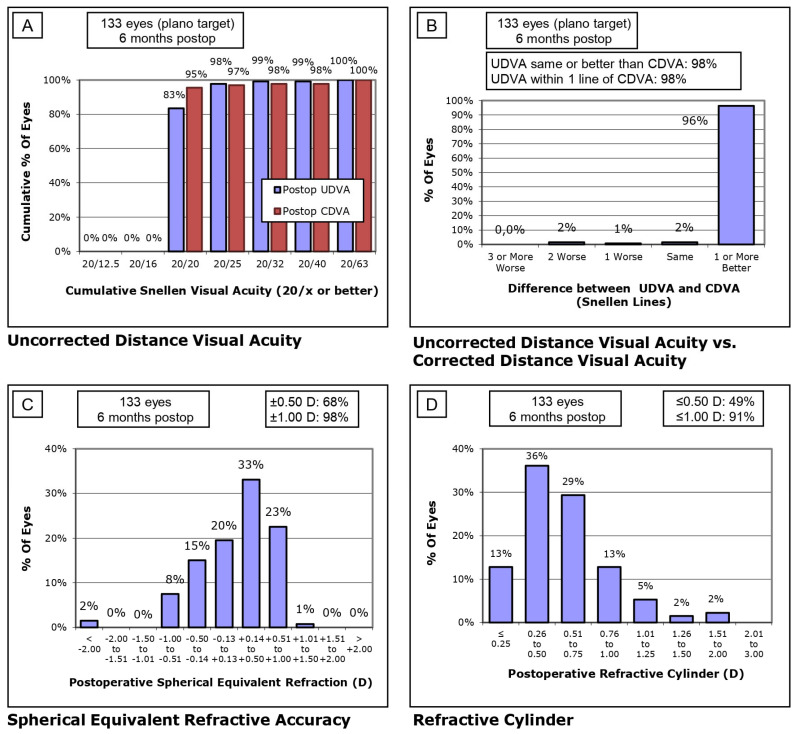
Standard visual and refractive outcomes of SS-OCT. **(A**) Uncorrected Distance Visual Acuity (UDVA): Cumulative Snellen visual acuity results, (**B**) Comparison between UDVA and Corrected Distance Visual Acuity (CDVA), (**C**) Spherical Equivalent Refractive Accuracy and (**D**) Refractive Cylinder: Distribution of postoperative refractive cylinder.

**Figure 2:**
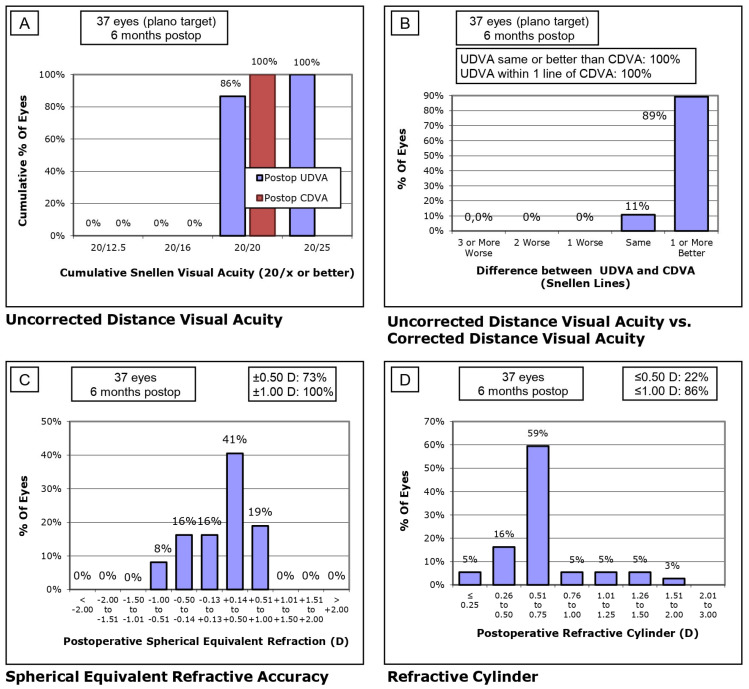
Standard visual and refractive outcomes of OLCI. (**A**) Uncorrected Distance Visual Acuity (UDVA): Cumulative Snellen visual acuity results, **(B**) Comparison between UDVA and Corrected Distance Visual Acuity (CDVA), (**C)** Spherical Equivalent Refractive Accuracy and **(D**) Refractive Cylinder: Distribution of postoperative refractive cylinder.

Figure 1 (SS-OCT group) and Figure 2 (OLCI group) illustrate the postoperative visual and refractive outcomes at 6 months, analyzed across four sections (Figure 1A–D and Figure 2A–D). In the SS-OCT group (Figure 1A), 98% of eyes achieved a UDVA of 20/20 or better, with a steep cumulative curve indicating excellent outcomes. In the OLCI group (Figure 2A), 100% of eyes reached 20/20 or better, demonstrating optimal uncorrected visual performance in this cohort. Both groups reflect excellent refractive targeting, with slightly higher precision observed in the OLCI group. The difference between UDVA and CDVA is minimal in both groups (Figure 1B and Figure 2B). In the SS-OCT group, 96% of eyes achieved the same or better UDVA compared to CDVA. Similarly, in the OLCI group, 100% of eyes achieved the same or better UDVA compared to CDVA. This highlights the strong visual outcomes achieved with both technologies. Refractive accuracy was high in both groups (Figure 1C and Figure 2C). In the SS-OCT group, 98% of eyes were within ±1.00 D of the target refraction, with 68% within ±0.50 D. In the OLCI group, 100% of eyes were within ±1.00 D, with a notably higher proportion, 73%, achieving ±0.50 D. These results reflect superior fine refractive precision in the OLCI group. Astigmatism correction was effective in both groups (Figure 1D and Figure 2D). In the SS-OCT group, 49% of eyes had a refractive cylinder ≤0.50 D, and 91% ≤1.00 D, showing robust astigmatism management in a larger cohort. In the OLCI group, 22% of eyes achieved ≤0.50 D, and 86% ≤1.00 D, indicating slightly lower astigmatism correction effectiveness compared to SS-OCT.

## 4. Discussion

This study provides a comprehensive comparison of the predictive accuracy and refractive outcomes achieved with SS-OCT and OLCI biometry technologies in the context of cataract surgery. Both methods demonstrated high reliability and clinical applicability, with MAE and MedAE aligning well with established benchmarks in the literature, highlighting their precision in refractive predictions. Furthermore, the percentage of eyes within ±0.25 D, ±0.50 D, ±0.75 D, and ±1.00 D of the predicted SE was substantial for both technologies, underscoring their capacity to meet the expectations of modern cataract surgery for accurate refractive outcomes.

### 4.1. Comparison of Refractive Accuracy with SS-OCT-Based Studies

The refractive accuracy achieved using SS-OCT technology in our study aligns with the high standards reported in the existing literature. SS-OCT biometers, known for their advanced imaging capabilities and precise measurements, consistently demonstrate superior predictability in cataract surgery outcomes.

Cheng et al. [18] reported that 97% of eyes achieved a SE within ±1.00 D and 78% within ±0.50 D using the IOLMaster 700 (Carl Zeiss Meditec, Jena, Germany). This reflects the capability of SS-OCT to deliver precise refractive outcomes, particularly in complex cases involving toric IOLs. Similarly, Kato et al. [19] found that 96% of eyes achieved SE within ±1.00 D using the Argos (MOVU Inc., Komaki, Japan), though they noted challenges in cases requiring significant astigmatic correction. Gledrum et al. [1] achieved comparable results, with 94% of eyes within ±1.00 D and 70% within ±0.50 D using the Anterion (Heidelberg Engineering, Heidelberg, Germany). Their study underscored the excellent performance of SS-OCT biometers, even though variability in extreme axial lengths posed occasional challenges.

Savini et al. [20] demonstrated similar outcomes, achieving SE within ±1.00 D in 95% of eyes using the OA-2000 (Tomey Corp., Nagoya, Japan). This emphasized the reliability of SS-OCT in cases with dense cataracts, where traditional biometry methods often face limitations. Macalinden et al. [5] evaluated refractive accuracy in toric IOL implantation using the Eyestar 900 (Haag-Streit AG, Köniz, Switzerland) and achieved SE within ±1.00 D in 90% of eyes and ±0.50 D in 68% of eyes. While slightly lower than other reports, these findings reaffirm the high reliability of SS-OCT technology in astigmatism correction.

In our study, the SS-OCT group achieved SE within ±1.00 D in 98% of eyes and ±0.50 D in 68% of eyes. These results align closely with or surpass the outcomes reported in other studies. Additionally, 91% of eyes in our study achieved a refractive cylinder ≤1.00 D, demonstrating excellent astigmatism correction that is consistent with the findings of Gledrum et al. [1] and Macalinden et al. [5]. The even distribution of with-the-rule (WTR) and against-the-rule (ATR) astigmatism in our cohort further highlights the robust performance of SS-OCT biometry in addressing diverse astigmatic profiles.

The consistent performance across studies confirms the reliability of SS-OCT technology in achieving precise refractive outcomes. Our findings, with high rates of refractive accuracy and excellent astigmatism correction, further validate SS-OCT as a cornerstone of modern cataract surgery planning. These results underscore the importance of SS-OCT technology in achieving precise and predictable refractive outcomes, providing a valuable tool for optimizing surgical results.

### 4.2. Comparison of Refractive Accuracy with OLCI/OLCR-Based Studies

Refractive prediction accuracy using OLCI/OLCR technologies demonstrates their reliability in achieving optimal postoperative outcomes, especially in cases where advanced biometry is critical. Studies utilizing OLCI/OLCR biometers, such as the Lenstar LS 900 (Haag-Streit AG, Köniz, Switzerland) and Aladdin (Topcon Corp., Tokyo, Japan), report consistent and precise refractive outcomes, underscoring their utility in cataract surgery planning.

Hoffer et al. [4] evaluated the refractive prediction accuracy of the Lenstar LS 900, reporting 93% of eyes achieving a SE within ±1.00 D and 72% within ±0.50 D. These findings highlight the device’s precision, particularly in calculating IOL power for eyes with regular corneal astigmatism. Similarly, Reitblat et al. [21] reported SE accuracy within ±1.00 D in 92% of eyes and ±0.50 D in 69% using the Lenstar LS 900. Their study emphasized the importance of robust biometry in challenging cases, including short axial lengths.

Shammas et al. [9] examined outcomes using the Aladdin, achieving SE within ±1.00 D in 90% of eyes and ±0.50 D in 67% of eyes. They observed slightly higher variability in extreme axial lengths and in cases requiring toric IOLs, which reflects the importance of lens constant optimization. Song et al. [22] found similar outcomes with the Lenstar LS 900, with 94% of eyes achieving SE within ±1.00 D and 70% within ±0.50 D. Their study highlighted the strength of OLCR technology in providing consistent and predictable outcomes across diverse patient cohorts.

In our study, the OLCI group achieved SE within ±1.00 D in 100% of eyes and ±0.50 D in 73% of eyes, demonstrating exceptional refractive prediction accuracy. Compared to the SS-OCT group, the OLCI cohort exhibited slightly higher precision, with a greater proportion of eyes achieving outcomes within closer refractive targets. Additionally, 86% of eyes in the OLCI group achieved a refractive cylinder ≤1.00 D, a result consistent with findings reported by Shammas et al. [9] and Song et al. [22]. This level of precision reinforces the effectiveness of OLCI technology in managing astigmatism during cataract surgery. Overall, the refractive accuracy achieved with OLCI/OLCR biometers confirms their high reliability and precision in modern cataract surgery.

### 4.3. Limitations

This study has several limitations that should be acknowledged. First, its retrospective design introduces inherent biases, including selection bias and a lack of control over confounding variables. Retrospective analyses rely on pre-existing clinical data, which may not always be comprehensive or standardized, potentially affecting the robustness of the findings. Second, the study was conducted at a single center with a small sample size, which may limit the generalizability of the results to other populations and clinical settings. Future multicenter studies with larger cohorts are needed to validate these findings.

Third, the follow-up period of six months, although sufficient to evaluate early and intermediate outcomes, is inadequate to assess long-term stability, durability of refractive outcomes, and late-onset complications, such as regression, corneal ectasia, or changes in higher-order aberrations. Extended follow-up periods are necessary to determine the persistence of the observed benefits.

Fourth, while a comparative group was included, the study did not evaluate the full spectrum of potential differences in surgical approaches or devices, limiting broader interpretations of the outcomes. Comprehensive comparative analyses with a wider range of surgical techniques and technologies could provide additional insights into the advantages and limitations of the studied intervention.

Fifth, there was an imbalance in sample sizes between the SS-OCT and OLCI groups due to clinical workflow constraints. To address this, we applied bootstrapping techniques to ensure result robustness. Future studies with larger, balanced cohorts are recommended for validation.

Lastly, the study excluded patients with irregular corneas or significant ocular comorbidities, which restricts the applicability of the findings to a more diverse patient population. Additionally, the study did not address potential socioeconomic factors or patient-reported outcomes, such as satisfaction or quality of life, which are increasingly recognized as important measures in refractive surgery research.

### 4.4. Future Lines of Research

Future research should focus on extending follow-up periods beyond six months to evaluate long-term stability and potential late-onset complications. Comparative studies with other advanced surgical techniques and technologies are needed to highlight the advantages and limitations of the current procedure. Additionally, its efficacy in treating complex refractive errors, such as high myopia, hyperopia, or irregular corneas, should be explored. Incorporating advanced imaging technologies and AI-based planning could further enhance treatment precision. Finally, patient-reported outcomes, including satisfaction and quality of vision under various conditions, should be systematically evaluated to provide a holistic understanding of the procedure’s impact.

### 4.5. Clinical Application

This study underscores the value of advanced biometry technologies, particularly SS-OCT and OLCI, in cataract surgery and refractive planning. SS-OCT biometers, such as Argos^®^, excel in precise AL and keratometry measurements, crucial for challenging cases like dense cataracts. OLCI biometers, such as Aladdin^®^, offer consistent anterior segment measurements for routine clinical practice. Both devices demonstrated high accuracy in predicting refractive outcomes, minimizing postoperative surprises.

The precision in keratometry supports accurate alignment of toric IOLs, improving visual outcomes and patient satisfaction. These findings reinforce the role of advanced biometers in enhancing the safety, predictability, and personalization of cataract and refractive surgery.

## 5. Conclusions

This study demonstrates that both SS-OCT and OLCI technologies provide reliable and accurate biometry for cataract surgery, with comparable refractive outcomes in most parameters. The superior performance of SS-OCT in specific aspects, such as axis alignment, underscores its potential for enhanced surgical precision. These findings support the continued use and development of advanced biometry technologies to optimize refractive outcomes in cataract surgery.

## Figures and Tables

**Table 1:** Preoperative characteristics of the study population.

Variable (Units)	SS-OCT	OLCI	*p*-Value
Age (years)	68.68 ± 11.28 (40–86)	69.57 ± 9.24 (53–86)	0.366
Sphere (D)	0.42 ± 3.13 (−12.00 to 8.00)	0.66 ± 2.79 (−8.00 to 4.50)	0.363
Cylinder (D)	−0.10 ± 1.62 (−4.50 to 4.25)	−0.08 ± 1.10 (−2.50 to 1.25)	0.481
Axis (degrees)	80.41 ± 60.72 (0–180)	82.07 ± 60.29 (3–180)	0.460
SE (D)	0.34 ± 3.34 (−13.13 to 8.63)	0.45 ± 2.42 (−8.00 to 4.50)	0.423
CDVA (LogMAR)	0.37 ± 0.31 (1.70 to 0.00)	0.44 ± 0.32 (1.00 to 0.00)	0.112
CED (cells/mm^2^)	2416.69 ± 304.43 (1442–3072)	2355.00 ± 293.86 (1602–2872)	0.140
IOP (mmHg)	16.06 ± 3.30 (10.0–27.5)	15.64 ± 3.92 (10.0–27.0)	0.258
Flat keratometry (D)	43.44 ± 1.63 (38.24–46.84)	43.48 ± 1.70 (39.64–47.67)	0.454
Steep keratometry (D)	44.49 ± 1.68 (39.68–48.16)	44.29 ± 1.63 (40.73–48.61)	0.267
Axis (degrees)	89.53 ± 57.25 (0–179)	104.39 ± 48.47 (10–178)	0.078
J0 (D)	−0.04 ± 0.39 (−0.94 to 1.51)	0.03 ± 0.28 (−0.58 to 1.18)	0.429
J45 (D)	−0.08 ± 0.44 (−1.55 to 1.65)	0.02 ± 0.33 (−0.90 to 1.01)	0.081
Lens Thickness (mm)	4.61 ± 0.42 (3.52–5.85)	4.76 ± 0.31 (4.21–5.42)	0.030
Axial Length (mm)	23.37 ± 1.23 (20.57–28.31)	23.17 ± 0.82 (21.71–24.82)	0174
ACD (mm)	3.23 ± 0.42 (2.01–4.02)	3.04 ± 0.40 (2.50–4.20)	0.009
WTW (mm)	11.93 ± 0.40 (10.94–12.96)	12.03 ± 0.50 (11.27–14.12)	0.117

SE: Spherical Equivalent, CDVA: Corrected Distance Visual Acuity, CED: Central Endothelial Density, IOP: Intraocular Pressure, J0: Jackson Cross Cylinder 0° component, J45: Jackson Cross Cylinder 45° component, ACD: Anterior Chamber Depth, and WTW: White to White.

**Table 2:** Surgical data, IOL power calculations, and refractive predictive accuracy.

Variable (Units)	SS-OCT	OLCI	*p*-Value
IOL Spherical Power (D)	21.83 ± 3.29 (10.50–33.00)	22.08 ± 1.77 (19.00–26.00)	0.332
IOL Astigmatic Power (D)	1.56 ± 0.98 (0.00–4.50)	2.00 ± 1.27 (1.00–3.75)	0.089
IOL Axis (degrees)	56.84 ± 66.94 (0–179)	59.50 ± 75.62 (2–179)	0.452
Suggested IOL Spherical Power (D)	21.68 ± 3.34 (10.00–33.00)	22.12 ± 1.90 (18.50–26.00)	0.221
Suggested IOL Astigmatic Power (D)	1.76 ± 0.83 (0.00–4.50)	1.97 ± 0.97 (1.00–3.75)	0.174
Suggested IOL Axis (degrees)	62.10 ± 67.45 (0–179)	90.00 ± 80.18 (2–190)	0.069
Difference Implanted vs. Suggested Power (Spherical, D)	0.15 ± 0.25 (−0.50 to 1.00)	−0.04 ± 0.61 (−2.50 to 0.50)	0.002
Difference Implanted vs. Suggested Power (Astigmatic, D)	−0.05 ± 0.35 (−3.00 to 1.00)	−0.27 ± 0.41 (−1.25 to 0.00)	0.035
Difference Implanted vs. Suggested Axis (degrees)	−3.98 ± 25.39 (−179 to 0)	−32.50 ± 76.59 (−180 to 15)	0.136
Expected Spherical Equivalent (D)	−0.16 ± 0.37 (−2.81 to 1.13)	−0.02 ± 0.36 (−0.47 to 1.25)	0.020
Mean Error 1-month vs. Expected SE (D)	0.33 ± 0.48 (−1.08 to 1.46)	0.24 ± 0.54 (−1.25 to 1.11)	0.181
Mean Error 6-months vs. Expected SE (D)	0.34 ± 0.41 (−0.92 to 1.45)	0.25 ± 0.49 (−1.25 to 1.21)	0.156
Mean Absolute Error 1-month vs. Expected SE (D)	0.47 ± 0.34 (0.01 to 1.46)	0.46 ± 0.35 (−0.92 to 1.45)	0.474
Mean Absolute Error 6-months vs. Expected SE (D)	0.44 ± 0.31 (0.00 to 1.45)	0.48 ± 0.26 (0.11 to 1.25)	0.206
Median Absolute Error 1-month vs. Expected SE (D)	0.41 (0.53) (0.01 to 1.46)	0.41 (0.65) (−0.92 to 1.45)	0.860
Median Absolute Error 6-months vs. Expected SE (D)	0.37 (0.49) (0.00 to 1.45)	0.49 (0.34) (0.11 to 1.25)	0.317
SE Refractive Prediction ±0.25 D * Eyes (percentage of eyes)	35 (26.3%)	10 (27.8%)	-
SE Refractive Prediction ±0.50 D * Eyes (percentage of eyes)	75 (56.4%)	20 (55.6%)	-
SE Refractive Prediction ±0.75 D * Eyes (percentage of eyes)	110 (82.7%)	30 (83.3%)	-
SE Refractive Prediction ±1.00 D * Eyes (percentage of eyes)	130 (97.7%)	35 (97.2%)	-

IOL: Intraocular Lens, SE: Spherical Equivalent. * Calculated based on ME of 6 months vs. expected SE.

**Table 3:** Postoperative outcomes at 1 and 6 months.

Variable (Units)	SS-OCT	OLCI	*p*-Value
IOP (mmHg)	14.03 ± 3.00 (8.0–23.0)	14.47 ± 2.56 (9.0–22.0)	0.211
Sphere 1-month (D)	0.24 ± 0.50 (−3.00 to 1.00)	0.32 ± 0.38 (−0.50 to 1.00)	0.172
Cylinder 1-month (D)	−0.15 ± 0.75 (−1.75 to 1.50)	−0.20 ± 0.64 (−1.25 to 1.00)	0.351
Axis 1-month (degrees)	89.09 ± 53.91 (1–180)	90.76 ± 60.90 (0–180)	0.440
SE 1-month (D)	0.14 ± 0.59 (−3.88 to 1.38)	0.18 ± 0.46 (−0.88 to 1.25)	0.256
UDVA 1-month (LogMAR)	0.03 ± 0.09 (0.90 to 0.00)	0.02 ± 0.03 (0.10 to 0.00)	0.157
CDVA 1-month (LogMAR)	0.02 ± 0.12 (0.90 to 0.00)	0.00 ± 0.00 (0.00–0.00)	0.563
Sphere 6-months (D)	0.22 ± 0.47 (−2.75 to 1.00)	0.36 ± 0.40 (−0.50 to 1.00)	0.053
Cylinder 6-months (D)	−0.07 ± 0.55 (−1.75 to 1.00)	−0.26 ± 0.63 (−1.75 to 1.00)	0.048
Axis 6-months (degrees)	78.70 ± 57.91 (0–180)	92.53 ± 54.60 (0–180)	0.117
SE 6-months (D)	0.15 ± 0.51 (−3.00 to 1.13)	0.19 ± 0.43 (−0.75 to 1.00)	0.339
UDVA 6-months (LogMAR)	0.02 ± 0.09 (0.90 to 0.00)	0.01 ± 0.02 (0.05 to 0.00)	0.182
CDVA 6-months (LogMAR)	0.02 ± 0.12 (0.90 to 0.00)	0.00 ± 0.00 (0.00–0.00)	0.157

IOP: Intraocular Pressure, SE: Spherical Equivalent, UDVA: Uncorrected Distance Visual Acuity, LogMAR: Logarithm of the Minimum Angle of Resolution, CDVA: Corrected Distance Visual Acuity.

## Data Availability

The data supporting the findings of this study are available from the corresponding author upon reasonable request.

## Abbreviations

The following abbreviations are used in this manuscript:

ACD	Anterior Chamber Depth
AL	Axial Length
CDVA	Corrected Distance Visual Acuity
D	Diopters
IOL	Intraocular Lens
logMAR	Logarithm of the Minimum Angle of Resolution
MAE	Mean Absolute Error
MedAE	Median Absolute Error
ME	Mean Error
OLCI	Optical Low-Coherence Interferometry
SE	Spherical Equivalent
SS-OCT	Swept-Source Optical Coherence Tomography
UDVA	Uncorrected Distance Visual Acuity
WTW	White-to-White Corneal Diameter
